# Supplementary figures and images for: Genetic adaptation to amoxicillin in Escherichia coli: The limited role of dinB and katE
Source: PLoS One. 2025 Feb 19;20(2):e0312223. doi: 10.1371/journal.pone.0312223 (PMC11838884; doi:10.1371/journal.pone.0312223)

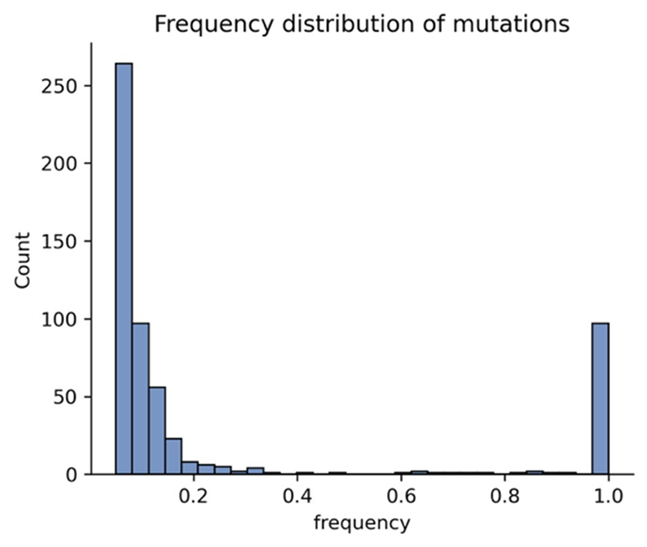

Supplement: S1 Fig — The graph illustrates the frequency distributions of mutations across the samples. (TIF) [file pone.0312223.s001.tif]
