# Supplementary material for: Genetic adaptation to amoxicillin in Escherichia coli: The limited role of dinB and katE
Source: PLoS One. 2025 Feb 19;20(2):e0312223. doi: 10.1371/journal.pone.0312223 (PMC11838884; doi:10.1371/journal.pone.0312223)
Supplement: S1 Table — (DOCX) [file pone.0312223.s002.docx]

| **Frequency** | **Sample** | **Position** | **Gene** | **New sequence** | **Type** |
| --- | --- | --- | --- | --- | --- |
| 1 | *ΔdinB* | coding (263/336 nt) | *prlF* | CATTCAA | INS |
| 1 | MG1655 | coding (263/336 nt) | *prlF* | CATTCAA | INS |
