# Supplementary material for: Genetic adaptation to amoxicillin in Escherichia coli: The limited role of dinB and katE
Source: PLoS One. 2025 Feb 19;20(2):e0312223. doi: 10.1371/journal.pone.0312223 (PMC11838884; doi:10.1371/journal.pone.0312223)
Supplement: S2 Table — (DOCX) [file pone.0312223.s003.docx]

| Sample number | Strain | Susceptible/Evolved | Antibiotic concentration [μg/mL] |
| --- | --- | --- | --- |
| 1 | MG1655 | Susceptible | - |
| 2 | MG1655 | Susceptible | - |
| 3 | MG1655 | Susceptible | 4 |
| 4 | MG1655 | Susceptible | 4 |
| 5 | MG1655 | Susceptible | 4 |
| 6 | MG1655 | Evolved | - |
| 7 | MG1655 | Evolved | - |
| 8 | MG1655 | Evolved | 1024 |
| 9 | MG1655 | Evolved | 1024 |
| 10 | Δ*dinB* | Susceptible | - |
| 11 | Δ*dinB* | Susceptible | - |
| 12 | Δ*dinB* | Susceptible | 4 |
| 13 | Δ*dinB* | Susceptible | 4 |
| 14 | Δ*dinB* | Susceptible | 4 |
| 15 | Δ*dinB* | Evolved | - |
| 16 | Δ*dinB* | Evolved | - |
| 19 | Δ*katE* | Susceptible | - |
| 20 | Δ*katE* | Susceptible | - |
| 21 | Δ*katE* | Susceptible | 4 |
| 22 | Δ*katE* | Susceptible | 4 |
| 23 | Δ*katE* | Susceptible | 4 |
| 24 | Δ*katE* | Evolved | - |
| 25 | Δ*katE* | Evolved | - |
| 26 | Δ*katE* | Evolved | 1024 |
| 27 | Δ*katE* | Evolved | 1024 |
| 30 | Δ*dinB* | Evolved | 512 |
| 31 | Δ*dinB* | Evolved | 512 |
| 32 | Δ*dinB* | Evolved | 512 |
