# Supplementary material for: Genetic adaptation to amoxicillin in Escherichia coli: The limited role of dinB and katE
Source: PLoS One. 2025 Feb 19;20(2):e0312223. doi: 10.1371/journal.pone.0312223 (PMC11838884; doi:10.1371/journal.pone.0312223)
Supplement: Attachment — Submitted filename: REVIEW REPORT_PONE-D-24-43795.pdf [file pone.0312223.s004.pdf]

REVIEW REPORT_PONE-D-24-43795

Dear Editor,

Thank you for providing me the opportunity to review this manuscript titled, **“Genetic Adaptation to Amoxicillin in *Escherichia coli*: The Limited Role of dinB and katE”** for publication in your journal (PLOS ONE). I have gone through the manuscript draft and found it quite interesting. The paper is clear and well-composed. The dataset therein is very useful. There is sufficient justification to affirm the originality of the work, numerical figures of the various analytical factors have been reported with a reasonable degree of accuracy. The objectives of the work as outlined in the introductory part of the manuscript have been reasonably achieved. Thus, the work could be adjudged complete within its limitations. In a nutshell, this is a high-quality manuscript that clarified the contributions of DNA polymerase IV (dinB) and catalase HP2 (katE) genes in *E. coli*'s adaptation to beta-lactam resistance. In particular, the work has established that dinB and katE may not be as critical for beta-lactam adaptation as generally assumed. This could be quite thought-provoking to the scientific community However; I have outlined some comments that could help improve the quality of the manuscript.

Comments:

1. Line 155. There may be no need of a hyphen between 5 and minute, also the word ‘minutes’ should be written in the plural form, to make it consistent with the other usage elsewhere in the manuscript.
2. L192. The concentration of the antibiotic used needs to be specified.
3. L196. Specify the concentration of the agarose used
4. L439 - 440 The direct involvement of *frd*D should be further elaborated to adequately discuss its role based on the findings of this study.
