# Supplementary material for: Genetic adaptation to amoxicillin in Escherichia coli: The limited role of dinB and katE
Source: PLoS One. 2025 Feb 19;20(2):e0312223. doi: 10.1371/journal.pone.0312223 (PMC11838884; doi:10.1371/journal.pone.0312223)
Supplement: Attachment — Submitted filename: Teichmann et al., response to the reviewer.docx [file pone.0312223.s005.docx]

Response to the reviewer PONE-D-24-43795

Teichmann et al.,: Genetic Adaptation to Amoxicillin in *Escherichia coli*: The Limited Role of dinB and katE

**Reviewers comments:**
Thank you for providing me the opportunity to review this manuscript titled, **“Genetic Adaptation to Amoxicillin in *Escherichia coli*: The Limited Role of dinB and katE”** for publication in your journal (PLOS ONE). I have gone through the manuscript draft and found it quite interesting. The paper is clear and well-composed. The dataset therein is very useful. There is sufficient justification to affirm the originality of the work, numerical figures of the various analytical factors have been reported with a reasonable degree of accuracy. The objectives of the work as outlined in the introductory part of the manuscript have been reasonably achieved. Thus, the work could be adjudged complete within its limitations. In a nutshell, this is a high-quality manuscript that clarified the contributions of DNA polymerase IV (dinB) and catalase HP2 (katE) genes in *E. coli'*s adaptation to beta-lactam resistance. In particular, the work has established that dinB and katE may not be as critical for beta-lactam adaptation as generally assumed. This could be quite thought-provoking to the scientific community However; I have outlined some comments that could help improve the quality of the manuscript.

**Comments:**

1. Line 155. There may be no need of a hyphen between 5 and minute, also the word ‘minutes’ should be written in the plural form, to make it consistent with the other usage elsewhere in the manuscript.
   Accepted and corrected accordingly
2. L192. The concentration of the antibiotic used needs to be specified.
   Accepted and corrected accordingly
3. L196. Specify the concentration of the agarose used
   Accepted and corrected accordingly
4. L439 - 440 The direct involvement of *frd*D should be further elaborated to adequately discuss its role based on the findings of this study.
   Frankly, we found this comments slightly confusing. The Involvement of *frd*D is extensively discussed in line 414-46. The combined authors came up with the following ideas to address this comment:
   1. "*Mutations in the frd operon have been described to boost ampC amplification and contribute to beta-lactam resistance”: Here, I guess we could compare the expression of ampC in strains with and without these mutations to add to the data in the cited papers*
   These data turned out very hard to compare, confusing instead of clarifying the issue.

2. "*changes in energy availability and cellular redox status, influenced by frdD expression, may affect the efficiency of cell wall synthesis, and by extension, bacterial susceptibility to beta-lactam antibiotics.”: This is a long-shot, but could we look at expression of e.g. cell wall synthesis pathway genes in relation to the frdD expression? If we see an association, I guess that would strengthen this case, but this might be hard as I know energy metabolism is affected by a bunch of different things*.
This association didn’t materialize either.

3. *We already analysed the fumarate reductase/succinate dehydrogenase relationship in detail, so this cannot be expanded*.

4. "*Superoxide dismutase activity has been linked to antibiotic resistance by activating the stringent response, upregulating efflux pumps, downregulating of the outer membrane porin OmpF, and co-regulation of the multidrug-resistant locus mar”: Do we see these upregulations together with increased frdD levels in our data?*
Turns out that this is not the case either.

5. "*elevated superoxide levels can increase mutagenesis”: We have the transcriptomics data from the final strains. It might be interesting to compare the frdD levels at the mid-point to the number of mutations in the corresponding final evolved strains. However, this may be comparing apples to oranges*.
Indeed, when this analysis was tried, the outcome was an uninterpretable mess.

In short, since the involvement of *frd*D has already been discussed extensively in the framework oh the literature (lines 414-46), we have decided not to add to that. We hope to have convinced you that this is not out of disrespect for the reviewer or lack of will to make the effort.
